# Bottom-to-top: A case report for treating esophageal cervical spondylosis with giant osteophytes

**DOI:** 10.1097/MD.0000000000046795

**Published:** 2026-01-02

**Authors:** Lei Chen, Shineng Lin, Zhenyu Shi, Qinwen Ge, Ju Li, Jinmin Liu, Peijian Tong, Taotao Xu, Yiqing Ling

**Affiliations:** a Department of Orthopedics and Traumatology, The First Affiliated Hospital of Zhejiang Chinese Medical University (Zhejiang Provincial Hospital of Chinese Medicine), Hangzhou, Zhejiang Province, China.

**Keywords:** esophageal cervical spondylosis, giant osteophytes, surgical technique

## Abstract

**Rationale:**

Esophageal cervical spondylosis is a rare condition characterized by dysphagia due to anterior cervical osteophyte compression. Existing surgical methods carry risks of esophageal injury and recurrence. This study introduces a novel “bottom-to-top” technique designed to safely resect large, adherent osteophytes while preserving esophageal integrity.

**Patient concerns:**

A 67-year-old male presented with progressive dysphagia over one year, severely affecting food intake. His preoperative Eating Assessment Tool-10 score was 18, indicating significant swallowing impairment.

**Diagnoses:**

Imaging revealed significant anterior longitudinal ligament calcification and large osteophytes from C4 to C7, compressing the esophagus. The patient was diagnosed with esophageal cervical spondylosis after excluding gastrointestinal and other otorhinolaryngological disorders.

**Interventions:**

The patient underwent anterior C4/5 osteophyte resection, discectomy, and interbody fusion using the “bottom-to-top” approach, which involved resecting the osteophyte base first and progressing upward to minimize esophageal adhesion damage.

**Outcomes:**

Immediate postoperative improvement in dysphagia was observed. The Eating Assessment Tool-10 score decreased from 18 preoperatively to 0 at 1-year follow-up. Radiographs at 1 year showed satisfactory implant alignment without significant recurrence. At 3 years, mild neck stiffness and partial osteophyte regrowth were noted, but no recurrence of dysphagia occurred.

**Lessons:**

The “bottom-to-top” technique is effective for safely resecting large, adherent osteophytes in esophageal cervical spondylosis, reducing intraoperative esophageal injury and maintaining functional improvement. Long-term monitoring is essential due to the possibility of anatomical recurrence. This approach holds promise for similar challenging cases, though further studies are needed to optimize rehabilitation and prevent recurrence.

## 1. Introduction

Cervical spondylosis refers to a group of disorders caused by degenerative changes in the cervical intervertebral discs and subsequent pathological changes in adjacent structures. These changes may involve surrounding tissues such as nerves and blood vessels, leading to clinical manifestations consistent with imaging findings.^[1]^ Clinically, cervical spondylosis is typically classified into 5 types: cervical type, radiculopathy type, myelopathy type, vertebral artery type, and sympathetic type. Different therapeutic approaches, including conservative and surgical treatments, are adopted based on the specific type of cervical spondylosis.^[2,3]^

Esophageal cervical spondylosis is a rare and distinct subtype of cervical spondylosis, also known as Forestier disease, accounting for approximately 1.6 to 1.7% of all cases.^[4]^ This condition arises from calcification and ossification of the anterior longitudinal ligament, along with vertebral osteophyte formation, which compresses the esophagus, leading to dysphagia. It predominantly affects patients aged 45 years and older.^[5–8]^ Early symptoms may include swallowing difficulties, speech impairment, dyspnea, and localized pain or functional restriction of adjacent soft tissues.^[9]^ The primary treatment is surgical, often involving anterior cervical osteophyte resection combined with cervical discectomy and interbody fusion, which helps mitigate the risk of secondary disc herniation postoperatively.^[5]^

This article presents a case of a patient with significant osteophyte growth at the C4 and C5 level, resulting in impaired swallowing and eating function. The patient underwent a novel surgical treatment, achieving favorable outcomes over a 2-year follow-up period. This report discusses the surgical approach and its clinical significance.

## 2. Case report

### 2.1. Patient basic information

A 67-year-old male patient presented with a 1-year history of progressive dysphagia, which had significantly worsened over the past 4 months. Initially, the patient experienced difficulty swallowing without an obvious trigger and required water to assist in swallowing food. The symptoms were initially ignored but later aggravated, severely affecting food intake in the past months. Based on the Eating Assessment Tool-10 criteria, the patient’s score was 18 points. Upon admission, his vital signs were stable: *T* 37°C, *P* 87 bpm, *R* 19 breaths/min, blood pressure 138/68 mm Hg. Physical examination revealed restricted cervical spine flexion and extension with loss of the normal cervical curvature, but no other notable abnormalities.

**Figure 1.**
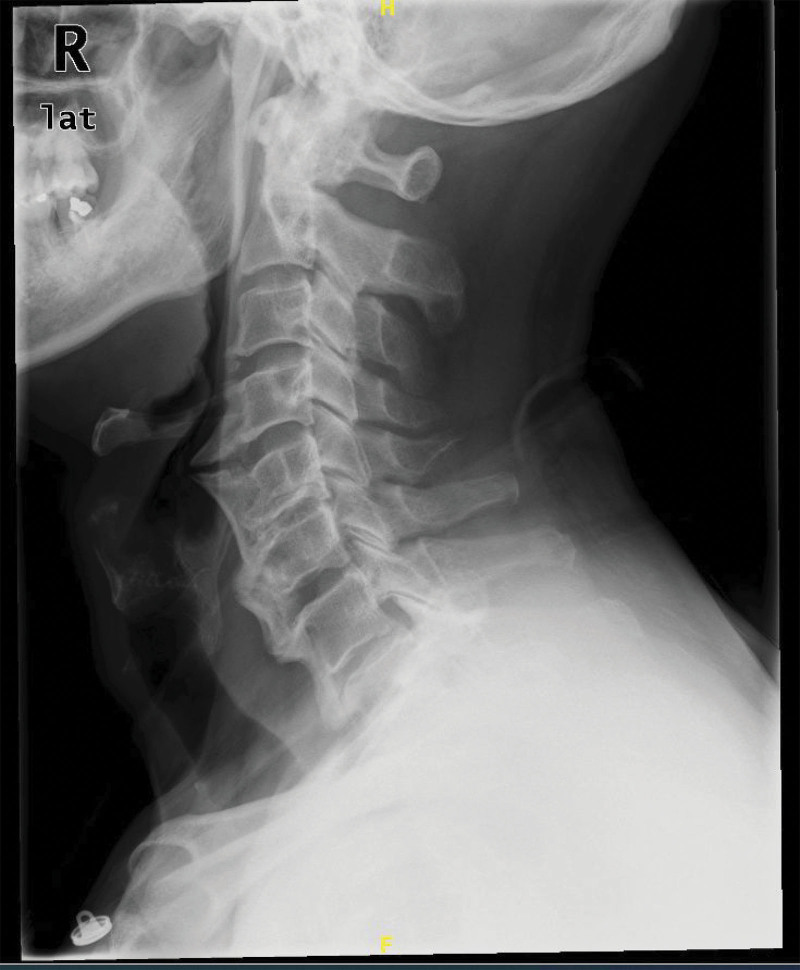
Preoperative lateral cervical X-ray showing significant calcification of the anterior longitudinal ligament and large osteophytes from C4 to C7 levels.

**Figure 2.**
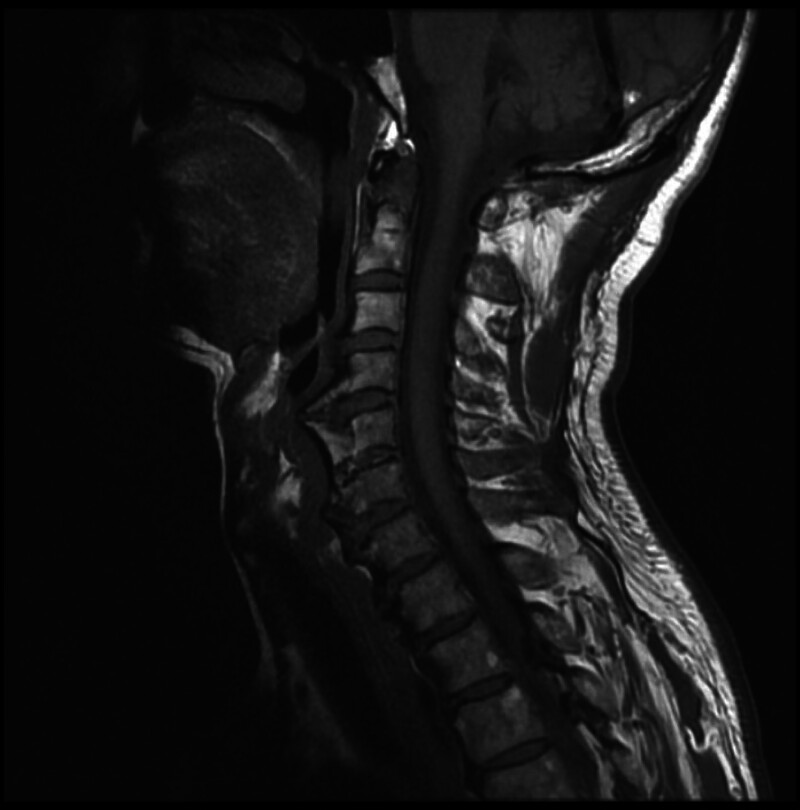
Preoperative T2-weighted MRI of the cervical spine demonstrating prominent anterior osteophytes at C4 to C7, causing compression and displacement of the esophagus. MRI = magnetic resonance imaging.

The patient had undergone laryngoscopy and other examinations at another hospital, which revealed no significant findings, ruling out digestive tract disorders. At our orthopedic clinic, imaging studies were conducted. X-rays revealed significant calcification of the anterior longitudinal ligament from C4 to C7 (Fig. 1). Magnetic resonance imaging (MRI) showed prominent anterior osteophyte formation from C4 to C7, partial formation of bony bridges, and narrowing of the C5 and C6 intervertebral space (Fig. 2). Although diffuse idiopathic skeletal hyperostosis (DISH) was initially considered, the diagnosis was excluded as the patient did not exhibit extra-spinal involvement, such as joint or enthesis pain and swelling, nor continuous ossification or calcification across 4 vertebral bodies with or without claw-like osteophytes, according to Resnick and Niwayama standard.^[10]^ Based on the clinical history and imaging findings, the patient was diagnosed with “esophageal cervical spondylosis.” After a comprehensive evaluation, the patient underwent anterior C4/5 osteophyte resection, C4/5 discectomy, and C4/5 interbody fusion on July 21, 2021.

### 2.2. Surgical procedure

The patient underwent general anesthesia and endotracheal intubation in a supine position with the neck elevated. Routine disinfection and draping were performed. C-arm fluoroscopy was used to locate and mark the C4/5 intervertebral space. A 4.5 cm horizontal incision was made on the right anterior cervical region, and subcutaneous tissue was dissected layer by layer with the assistance of an electric scalpel. The platysma was incised, and blunt dissection was performed along the medial edge of the sternocleidomastoid muscle to reach the prevertebral fascia. The carotid sheath was retracted laterally, and the esophagus and trachea were retracted to the opposite side to expose the vertebral body and anterior longitudinal ligament. Meticulous blunt dissection was performed to minimize traction on the esophagus and reduce the risk of injury to the recurrent laryngeal nerve. Intraoperative neuromonitoring was not employed; instead, careful anatomical dissection and avoidance of excessive retraction were the primary methods for nerve protection.

**Figure 3.**
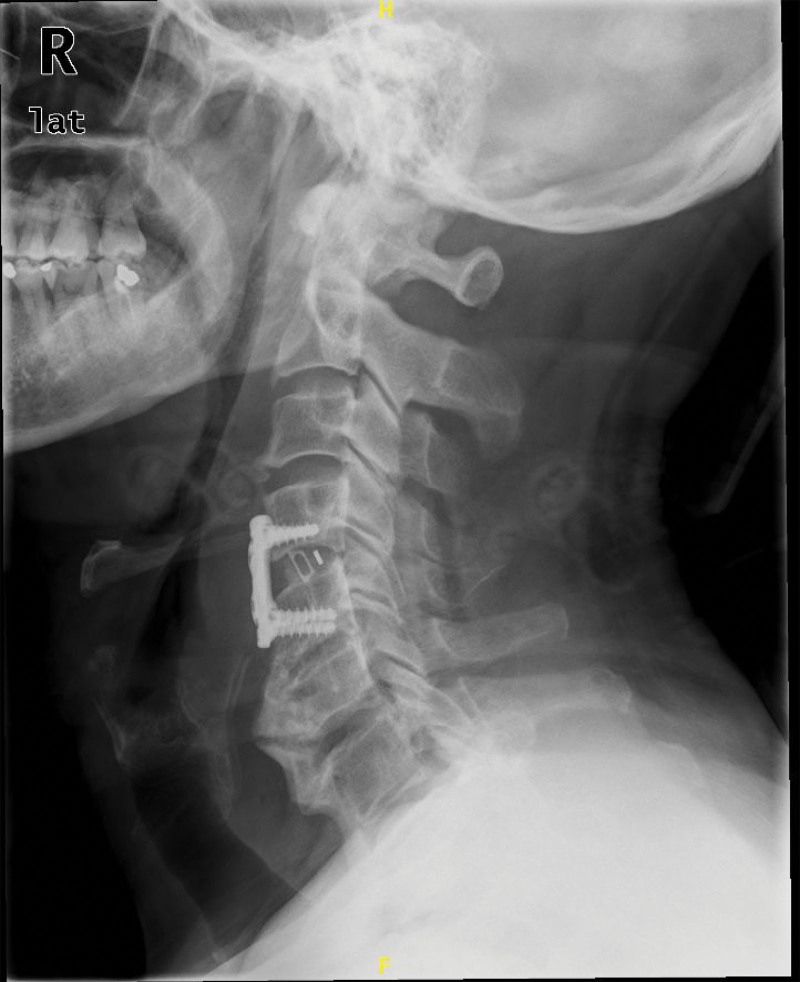
Immediate postoperative lateral cervical X-ray showing the implanted C4 and C5 interbody cage and anterior cervical plate with screws in satisfactory position.

Intraoperatively, a prominent beak-shaped osteophyte was observed at the anterior edge of the C4/5 vertebral body. The anterior longitudinal ligament was incised, and the osteophyte was removed incrementally using a rongeur. The “bottom-to-top” technique was employed: using a rongeur and subsequently a high-speed drill, resection began at the base of the osteophyte near the vertebral body and proceeded superiorly towards its tip. Osteophyte removal started at the base near the vertebral body and proceeded upward to minimize the risk of damaging the adhesions between the osteophyte and esophageal mucosa. After most of the osteophyte was resected, the anterior edge of the C4/5 vertebral body was reshaped to ensure stability for subsequent fixation. The C4/5 nucleus pulposus (approximately 1.5 × 1.5 cm) was removed using a nucleus pulposus clamp, and the inferior endplate of C4, the superior endplate of C5, and part of the bone tissue were scraped to expose the posterior longitudinal ligament. A No. 6 interbody cage was implanted, and an appropriate-length anterior cervical plate with screws was fixed at C4 and C5. The decision for interbody fusion was based on concerns regarding potential instability after extensive osteophyte resection and to mitigate the risk of osteophyte recurrence by reducing segmental mobility.^[11,12]^ The screw positions were confirmed as satisfactory under fluoroscopy. The surgical site was irrigated, hemostasis was achieved, and a drainage tube was placed. The incision was closed layer by layer, and a cervical collar was applied for immobilization. The patient was instructed to wear the cervical collar for 6 weeks postoperatively. Formal swallowing therapy was not initiated as the patient’s swallowing function improved rapidly post-surgery. You can see Figure 3.

**Figure 4.**
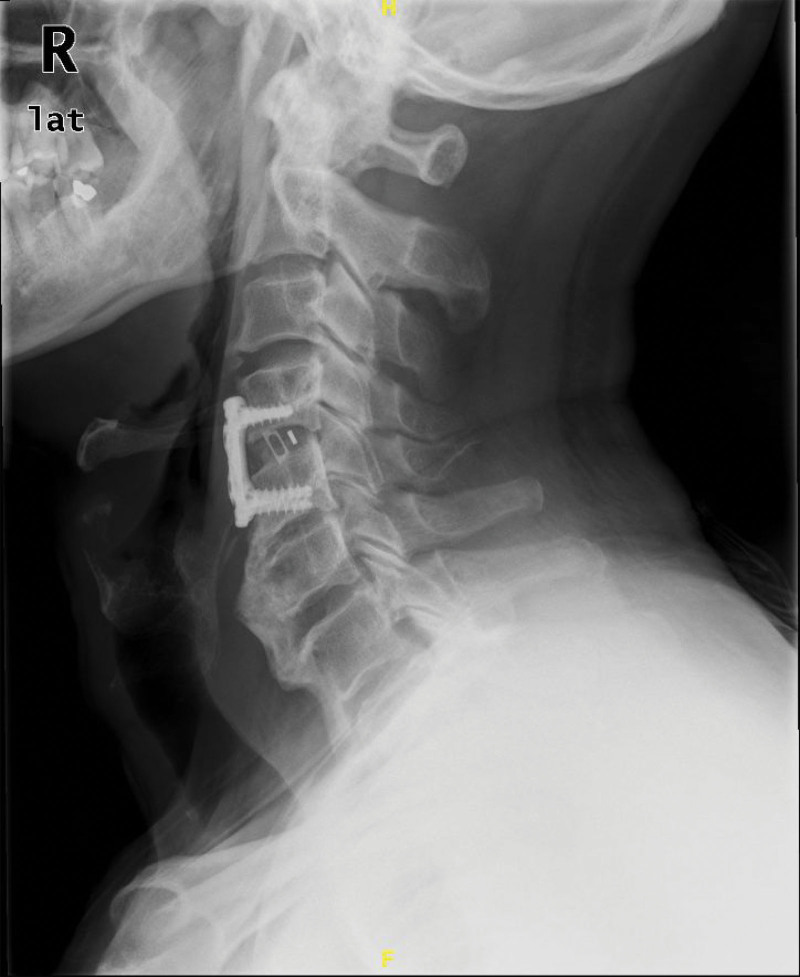
Lateral cervical X-ray at 1-year follow-up showing maintained alignment and instrumentation without significant osteophyte regrowth.

**Figure 5.**
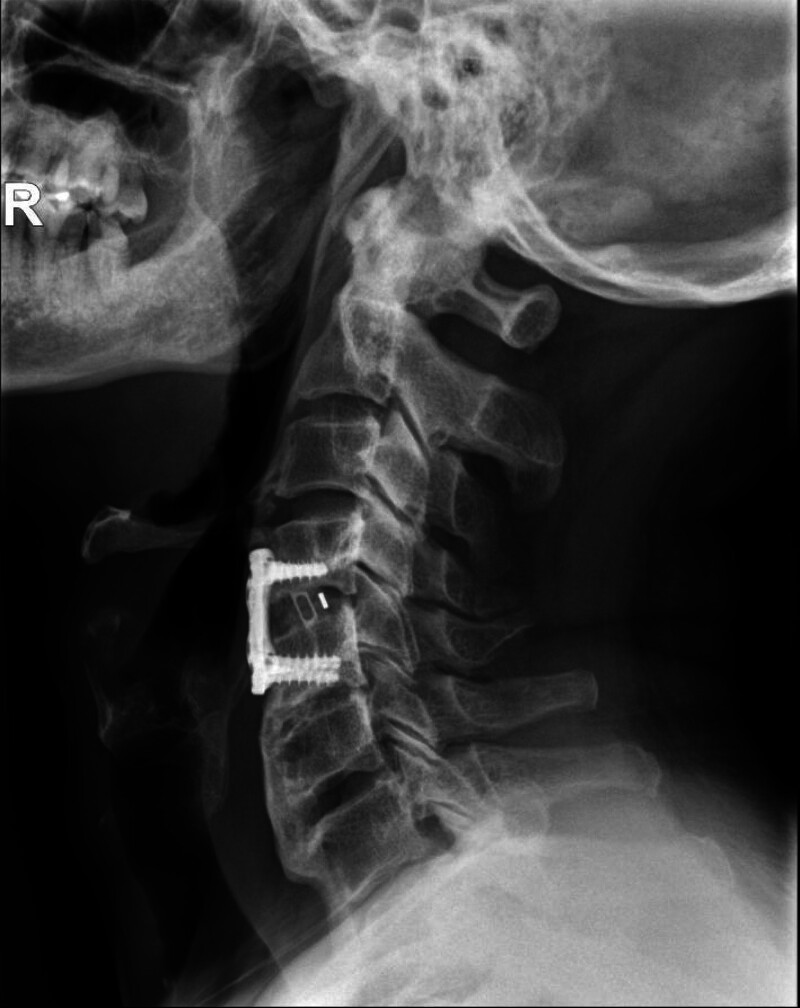
Lateral cervical X-ray at 3-year follow-up demonstrating partial regrowth of osteophytes along the anterior margins of C4 and C5.

At 1-year postoperative follow-ups, the patient exhibited significant improvement in dysphagia symptoms, with the Eating Assessment Tool-10 score decreasing from the preoperative level of 18 to 0, with no abnormalities observed on radiographic imaging. However, at the 3-year follow-up, the patient reported neck stiffness accompanied by mild pain, and X-rays revealed partial osteophyte growth along the vertebral margins. No deterioration was observed in the swallowing function (Figs. 4 and 5).

## 3. Discussion

### 3.1. Diagnosis of esophageal cervical spondylosis

Esophageal cervical spondylosis is a rare and distinct subtype of cervical spondylosis, characterized by esophageal compression caused by osteophyte formation and calcification of the anterior longitudinal ligament.^[5]^ Unlike the 5 traditional types of cervical spondylosis (cervical type, radiculopathy type, myelopathy type, vertebral artery type, and sympathetic type), its clinical manifestations primarily include dysphagia, a foreign body sensation in the throat, or dyspnea, rather than neurological dysfunction. Due to its unique clinical features, the incidence of this condition is limited to 1.6 to 1.7%.^[4]^ Its rarity, coupled with nonspecific early symptoms such as dysphagia, often leads to misdiagnosis as a gastrointestinal or otorhinolaryngological disorder, making the diagnosis particularly challenging.^[13]^ Systematic exclusion of other common causes of dysphagia, such as esophageal carcinoma, pharyngeal diverticula, or strictures, is crucial. In this case, laryngoscopy and previous gastrointestinal evaluations effectively ruled out these alternative diagnoses.

Currently, there is no molecular biological basis for diagnosing esophageal cervical spondylosis, and imaging examinations are the primary diagnostic tool. Simple lateral cervical spine X-rays can reveal osteophytes and anterior longitudinal ligament calcification. Additionally, differential diagnosis is essential to distinguish esophageal cervical spondylosis from other conditions, such as esophageal cancer, pharyngeal diverticulum, laryngeal tumors, and achalasia. In this case, the patient presented with stable vital signs and no significant positive findings during physical examination. The key findings were straightening of the cervical spine’s physiological curvature and limited neck mobility, with no apparent neurological deficits. Imaging studies, such as X-rays, are sufficient for a definitive diagnosis, while computed tomography and MRI provide detailed visualization of the relationship between osteophytes and surrounding soft tissues, aiding in the identification of complications and surgical planning. Furthermore, esophagography, gastrointestinal endoscopy, and laryngoscopy are valuable for excluding other gastrointestinal disorders.^[13]^

In this case, the patient initially underwent examinations at an external hospital, which ruled out gastrointestinal disorders, highlighting the potential for misdiagnosis of esophageal cervical spondylosis in clinical practice. At our orthopedic clinic, X-ray imaging revealed significant osteophyte growth, and subsequent MRI evaluation further clarified the condition of surrounding soft tissues, including the intervertebral disc, spinal canal, and esophageal compression, as well as the state of the trachea and epiglottis. These comprehensive imaging findings not only confirmed the diagnosis but also informed the development of an effective surgical treatment plan.

This case represents a typical instance of esophageal cervical spondylosis, demonstrating the diagnostic and differential diagnostic features of the condition. Compared with other reported cases, the osteophyte growth in this patient was more pronounced and represents one of the largest esophageal cervical spondylosis osteophytes reported in the literature, underscoring the classic nature and clinical significance of this case.

### 3.2. Etiology of esophageal cervical spondylosis

Esophageal cervical spondylosis is primarily caused by calcification of the anterior longitudinal ligament and vertebral body hypertrophy. However, its exact pathogenesis remains unclear. Current evidence suggests that DISH is the condition most closely associated with esophageal cervical spondylosis. Even so, the occurrence of dysphagia caused by anterior longitudinal ligament calcification in the cervical spine remains rare, even among patients with DISH. A recent systematic review identified age, obesity, and hypertension as potential risk factors for the development of this condition.^[14]^ At the molecular level, esophageal cervical spondylosis appears to be linked to genetic, metabolic, and vascular factors.^[13]^ Several studies have identified genes associated with the condition, such as FGF2, COL6A1, PPP2R2D, and BMP4. However, these findings are primarily derived from database or bioinformatics analyses, and no studies have established a direct relationship between these genes and specific phenotypic manifestations of esophageal cervical spondylosis or their influence on disease progression.^[15–18]^

Additionally, some research has indicated that DISH patients exhibit significantly increased vascularization and the size of the vertebral nutrient foramina,^[19]^ Bakker et al^[20]^ compared the morphology of thoracic and cervical osteophytes in DISH patients and found that the unrestricted forward growth of cervical osteophytes might be associated with the vascular structures in the anterior cervical region. Does this suggest that the development of esophageal cervical spondylosis, when induced by DISH, is linked to increased vascularization and the size of vertebral nutrient foramina? Could similar findings be observed in cases of esophageal cervical spondylosis caused by other conditions or occurring spontaneously? These questions remain unanswered and warrant further clinical investigation.

### 3.3. Treatment of esophageal cervical spondylosis

In its early stages, esophageal cervical spondylosis is often asymptomatic or presents with only mild symptoms.^[21]^ For mild-to-moderate cases, patients typically do not exhibit significant symptoms such as pain, upper limb numbness, or dizziness, and clinical intervention is usually not performed.^[11,12]^ Additionally, early diagnosis of such cases remains challenging, as no effective methods for early detection have been developed to date, highlighting a key direction for future research.

When osteophytes cause severe esophageal compression and dysphagia, surgical intervention becomes a necessary option. The primary goal of surgery is to remove the osteophytes, thereby relieving mechanical compression and restoring normal swallowing function. Regarding surgical options, the literature identifies 2 main approaches: anterior cervical osteophyte resection alone and osteophyte resection combined with interbody fusion. For severe cases of esophageal cervical spondylosis, osteophyte resection has been demonstrated to yield significant therapeutic benefits.^[4,5]^ In cases where cervical instability or spinal cord and nerve compression is present, combined interbody fusion is recommended.^[22]^ However, some studies have reported that simple osteophyte resection may reduce the cervical range of motion, leading to instability. Additionally, fusion surgery is recommended for patients under the age of 70 to reduce the risk of osteophyte recurrence. Based on these considerations, we performed anterior cervical osteophyte resection combined with interbody fusion for this patient to ensure immediate stability and potentially reduce the risk of recurrence by eliminating motion at the affected segment.^[11,12]^

For patients with large osteophytes, the surgical risk increases significantly due to potential adhesion between the osteophytes and surrounding structures, such as the anterior longitudinal ligament and fascia, which can heighten the risk of damage to critical tissues such as the esophagus and recurrent laryngeal nerve. In this case, based on preoperative imaging and intraoperative findings, we observed that the anterior edge of the osteophyte was tightly adhered to the esophagus. If the conventional approach of removing the anterior edge first had been adopted, it could have caused damage to the surrounding soft tissues.^[4]^ To mitigate this risk, we modified the surgical sequence, starting with resection of the osteophyte base and progressing forward to reshape the osteophyte tip. This “bottom-to-top” approach reduces the risk of esophageal injury by first destabilizing the osteophyte and allowing its adherent tip to be mobilized with minimal traction. By resecting the base, the leverage and contact area between the instrument and the adherent tip are reduced, decreasing the chance of tearing the esophageal wall during manipulation. Although this approach required more extensive exposure of the surgical field and placed higher demands on the surgeon’s skill, it effectively reduced the risk of intraoperative esophageal injury, thereby minimizing complications and postoperative recurrence rates. During a 3-year follow-up period, the patient demonstrated normal swallowing function without significant complications, further confirming that osteophyte resection is an effective treatment for improving symptoms of esophageal cervical spondylosis. Moreover, our novel “bottom-to-top” technique was shown to be highly effective in treating patients with large osteophytes tightly adhered to the esophagus, and its potential for broader clinical application warrants further exploration.

However, during the most recent follow-up, the patient reported neck stiffness, and imaging studies revealed marginal osteophyte growth. Several studies have documented osteophyte recurrence in esophageal cervical spondylosis within 15 months to 10 years following the initial surgery.^[23–25]^ This observation highlights the potential for postoperative recurrence, although its underlying mechanisms remain unclear. The recurrence mechanism may involve continued biomechanical stress, underlying systemic predisposition (even in non-DISH patients), or the natural history of the degenerative process. In our case, imaging at the 2-year follow-up revealed only mild marginal osteophyte growth, with no recurrence of dysphagia symptoms, possibly attributable to the simultaneous fusion and stabilization performed during the initial surgery. Further studies are needed to elucidate the mechanisms of recurrence and optimize postoperative management strategies.

### 3.4. Limitations

This study has several limitations. Firstly, it is a single case report, which limits the generalizability of the findings. The lack of a control group prevents direct comparison with other surgical techniques. Secondly, the assessment of swallowing improvement, though clinically significant, was primarily descriptive and based on patient report; the use of standardized, quantitative swallowing scales (e.g., videofluoroscopic swallowing study) would have provided more objective outcome measures. To prevent the recurrence of osteophyte growth, could we consider extending the duration of postoperative cervical brace fixation or implementing more comprehensive and appropriate rehabilitation measures? For instance, would avoiding excessive cervical flexion and extension postoperatively reduce the likelihood of recurrence? Currently, there is a lack of systematic research on rehabilitation strategies for such patients. Future cohort studies, if feasible, should incorporate specific rehabilitation interventions to evaluate their effectiveness in preventing osteophyte regrowth.

Furthermore, for patients experiencing recurrent esophageal cervical spondylosis, what surgical approaches could minimize the damage associated with reoperation while effectively reducing the risk of further recurrence? These questions are crucial for advancing the understanding and management of this rare and under-researched condition. Large-scale randomized controlled trials are urgently needed to address these challenges and provide evidence-based solutions.

## 4. Conclusion

This case provides a detailed exploration of the rarity and distinctive clinical features of esophageal cervical spondylosis, emphasizing the critical importance of early diagnosis. Imaging studies, particularly X-rays and MRI, are instrumental in identifying osteophyte formation and its compression of the esophagus, thereby facilitating the development of individualized treatment plans. For patients with mild symptoms, conservative treatment may be the preferred approach. However, in cases of severe symptoms, surgical intervention becomes necessary and effective.

This case highlights an innovative surgical approach that minimized damage to surrounding tissues while significantly improving the patient’s swallowing function. Nonetheless, postoperative monitoring remains essential to address potential osteophyte recurrence and the reemergence of related symptoms. In summary, the management of esophageal cervical spondylosis requires a comprehensive approach that integrates clinical presentation, imaging assessments, and individualized treatment strategies to ensure optimal therapeutic outcomes and improved patient prognosis.

## Acknowledgments

We thank all the individuals whose contributions and support have been invaluable throughout the process of writing this paper.

## Author contributions

**Conceptualization:** Lei Chen, Shineng Lin, Zhenyu Shi, Qinwen Ge, Ju Li, Jinmin Liu, Peijian Tong, Taotao Xu, Yiqing Ling.

**Data curation:** Lei Chen, Shineng Lin, Zhenyu Shi, Qinwen Ge, Ju Li, Jinmin Liu, Peijian Tong, Taotao Xu, Yiqing Ling.

**Formal analysis:** Lei Chen, Shineng Lin, Zhenyu Shi, Qinwen Ge, Ju Li, Jinmin Liu, Peijian Tong, Taotao Xu, Yiqing Ling.

**Funding acquisition:** Lei Chen.

**Investigation:** Lei Chen and Shineng Lin.

**Methodology:** Lei Chen, Shineng Lin, Zhenyu Shi, Qinwen Ge, Ju Li, Jinmin Liu, Peijian Tong, Taotao Xu, Yiqing Ling.

**Project administration:** Lei Chen.

**Resources:** Lei Chen.

**Software:** Lei Chen.

**Supervision:** Lei Chen.

**Validation:** Lei Chen.

**Visualization:** Lei Chen.

**Writing – original draft:** Lei Chen.

**Writing – review & editing:** Lei Chen, Shineng Lin, Zhenyu Shi, Qinwen Ge, Ju Li, Jinmin Liu, Peijian Tong, Taotao Xu, Yiqing Ling.
